# Risk Factors of Metabolic Syndrome in Community-Dwelling People with Schizophrenia

**DOI:** 10.3390/ijerph17186700

**Published:** 2020-09-14

**Authors:** Min Jung Sun, Mi Heui Jang

**Affiliations:** College of Nursing Science, Kyung Hee University, Seoul 02447, Korea; dnfntk0213@khu.ac.kr

**Keywords:** schizophrenia, metabolic syndrome, lifestyle, depression, social support

## Abstract

This study investigated the prevalence and risk factors of metabolic syndrome in 100 community-dwelling people with schizophrenia registered in mental health facilities in Seoul, Korea. This study was conducted between 12 September and 15 November 2019. This study used a cross-sectional descriptive design. The data included were general and disease-related characteristics, diagnostic tests for metabolic syndrome, lifestyles, depression, and social support. The analysis of collected data was done by using the SPSS 24.0 program. The prevalence of metabolic syndrome was 42.0%. Higher body mass index (odds ratio [OR] = 1.60, 95% CI = 1.16–2.18, *p* = 0.004), and depression (OR = 1.22, 95% CI = 1.06–1.42, *p* = 0.008) were associated with higher risks of metabolic syndrome, while physical activity and weight control (OR = 0.71, 95% CI = 0.54–0.94, *p* = 0.018), dietary habits (OR = 0.72, 95% CI = 0.54–0.93, *p* = 0.011), and medication and health management (OR = 0.52, 95% CI = 0.31–0.86, *p* = 0.012) were associated with lower risks. Mental health care nurses need to recognize the high prevalence of metabolic syndrome in people with schizophrenia in the community and provide differentiated, customized lifestyle improvement programs based on the body mass index and depression status of each person with schizophrenia. Furthermore, comprehensive lifestyle improvement programs and health examination services that people with schizophrenia can easily adhere to should be developed.

## 1. Introduction

Metabolic syndrome is a major public health concern, the prevalence of which is steadily increasing worldwide. This term refers to a cluster of metabolic disorders, including abdominal obesity, dyslipidemia, hypertension, and type 2 diabetes [1,2]. In people with schizophrenia, the prevalence of metabolic syndrome is two- to three-fold higher than that of the general population [3,4]. Metabolic disorders in people with schizophrenia increase their risk of developing cardiovascular disease, consequently reducing their life expectancy by approximately 10 to 25 years [5]. Therefore, the prevention and treatment of metabolic disorders are of critical importance.

Some of the reported risk factors that increase the likelihood of developing metabolic syndrome in schizophrenia are associated with disease-related characteristics and lifestyle choices [6,7]. In people with schizophrenia, negative symptoms, such as emotional blunting, anhedonia, avolition, and social withdrawal, interfere with their insights into physical activity and physical health. As a result, compared to the general population, they consume more readily available, high-fat, high-calorie fast foods and fewer fruits or vegetables, resulting in health problems such as vitamin deficiency and obesity [8]. Moreover, as the mental illness becomes chronic, affected individuals may maintain unhealthy lifestyle habits such as a prolonged lack of physical activity, smoking, and drinking, which predispose them to metabolic syndrome [4,9].

Antipsychotics used to alleviate the negative symptoms of schizophrenia have adverse effects, including increased appetite and delayed satiety [10]. In particular, clozapine and olanzapine cause metabolic disorders such as weight gain, type 2 diabetes, and cardiovascular disease [10,11]. Therefore, assessing and managing disease-related characteristics and the lifestyle habits of community-dwelling schizophrenia patients taking antipsychotics is an essential component of preventing the development of metabolic syndrome in these patients.

Psychosocial risk factors related to metabolic syndrome have been reported to include depression and social support [12]. Depression stimulates the hypothalamic–pituitary–adrenal axis to increase the secretion of cortisol, which induces visceral fat accumulation [13]. Depression also causes unhealthy habits, such as a lack of physical activity, unhealthy dietary patterns, excessive drinking, smoking, and sleeping problems, which contribute to the development of obesity and metabolic syndrome [14,15]. In a meta-analysis that confirmed the relationship between metabolic syndrome and depression, the prevalence of metabolic syndrome was 1.5 times higher in people with depression than in those without depression [15]. In people with schizophrenia, the prevalence of depression was reported to be 54.6%–61.0%, which is remarkably higher than that of the general population [16,17]. Judging from these results, we conjecture that metabolic syndrome in schizophrenia may be closely related to depression, and that management of depression could therefore address an important predisposing factor for metabolic syndrome. Previous studies have investigated the severity of metabolic syndrome in specific mental disorders, such as schizophrenia and major depressive disorder; however, there is a paucity of literature on the severity of metabolic syndrome in schizophrenia according to symptoms of depression, and further research would therefore provide useful insights into this issue.

As another psychosocial risk factor, social support plays a pivotal role in helping people with schizophrenia to initiate and maintain physical activity and provides other direct and indirect advantages in maintaining or improving health [18]. In previous studies involving participants without mental disorders, social support was also found to reduce depressive mood, increase physical activity, and promote healthy lifestyle modifications [19,20]. Furthermore, a study analyzing 81 previous publications on social support revealed that social support had beneficial, protective effects on the cardiovascular, endocrine, and immune systems, thereby reducing morbidity and mortality from the disease [21,22]. These previous studies allow us to predict that social support would also affect metabolic syndrome in people with schizophrenia, but this relationship has not yet been confirmed by studies.

According to a recent study, 85.5% of patients with mental disorders in Korea have comorbid physical health conditions such as cardiovascular disease, obesity, dyslipidemia, and diabetes [23]. However, although the majority of people with schizophrenia take their physical health problems seriously, they often do not receive proper treatment [24]. The lack of knowledge of physical health, social stigma, and lack of care from medical staff appear to be obstacles to the active monitoring of physical health in people with schizophrenia [25,26]. Although mental health care nurses in communities are in an ideal position to examine the physical health status of these patients, mental health care nurses have expressed that a lack of education and training can cause difficulties in managing patients’ physical health [27]. In addition, due to the lack of a systematic allocation of personnel and time to cover these tasks in the community mental health care centers in Korea, case management in Korea mostly focuses on psychiatric symptoms and medication management. The effectiveness of patient management is limited since physical health management depends on the competency of individual nurses [28]. Therefore, most chronic diseases—including metabolic syndrome—in people with chronic schizophrenia are managed ineffectively in a way that is highly individual-dependent.

In light of these problems, the purpose of this study was to identify the prevalence and risk factors of metabolic syndrome in community-dwelling people with schizophrenia and to establish a foundation for the development of nurse-led prevention and management programs targeting metabolic syndrome at community mental care health centers in the future. The specific objectives were as follows: First, to identify the general and disease-related characteristics, lifestyle habits, and degree of depression and social support of the participants; second, to identify the prevalence of metabolic syndrome among the participants; third, to investigate differences in general characteristics, disease-related characteristics, lifestyle habits, depression, and social support according to the presence or absence of metabolic syndrome; and fourth, to determine the risk factors for metabolic syndrome in the participants.

## 2. Materials and Methods

### 2.1. Study Design and Participants

This descriptive correlational study was conducted to investigate the prevalence and risk factors of metabolic syndrome in people with schizophrenia living in the community. This study was conducted between 12 September and 15 November 2019.

The participants of this study were people with schizophrenia in the community. The inclusion criteria of the study were as follows: 1st, having been diagnosed with schizophrenia according to the Diagnostic and Statistical Manual of Mental Disorders, Fifth Edition (DSM-5) by a psychiatrist; 2nd, being registered at 1 of 7 mental health welfare centers, 2 vocational rehabilitation facilities, 2 daytime rehabilitation facilities, or 1 reentry facility in Seoul, Korea; 3rd, being an adult aged 18–65 with no difficulties in communication and cooperation; and 4th, understanding the questionnaire and being able to communicate verbally and nonverbally. The exclusion criteria for the study were as follows: 1st, having neurological disorders other than psychiatric disorders or intellectual disability; 2nd, being addicted to alcohol or other drugs.

The number of participants was calculated using G*Power version 3.1.9.4 (Heinrich-Heine-Universität Düsseldorf, Düsseldorf, Germany). Based on a previous study [3] of factors associated with metabolic syndrome among people with schizophrenia, 82 participants were required, assuming an odds ratio of 2.26, a prH0 of 0.465, significance level 0.05, and power of 0.90. Data were gathered from 105 individuals, anticipating a 20% dropout rate. Data from 100 participants were used in the analysis, with the exclusion of 5 incomplete responses.

### 2.2. Measures

A demographic questionnaire was used to collect participants’ personal information, as well as information on their health and medical records. It gathered information on age, sex, educational background, job, income level, body mass index, duration of schizophrenia, and psychiatric medications.

Metabolic syndrome was measured by the American Heart Association/National Heart, Lung, and Blood Institute criteria, using Korean cut-offs for waist circumference [29]. According to these definitions, at least 3 out of 5 criteria must be fulfilled to diagnose metabolic syndrome. These criteria included elevated waist circumference (≥90 cm in males and ≥85 cm in females), elevated triglyceride levels (≥150 mg/dL or pharmacological treatment for elevated triglycerides), reduced high-density lipoprotein cholesterol (HDL-C) levels (<40 mg/dL in males and <50 mg/dL in females or pharmacological treatment for reduced HDL-C), elevated blood pressure (≥130/85 mmHg or pharmacological treatment for hypertension), and elevated fasting glucose levels (≥100 mg/dL or pharmacological treatment for elevated glucose).

Lifestyle was measured using Kang’s lifestyle evaluation tool [30]. This self-administered 36-item tool consisted of 6 domains: Physical activity and weight control (8 items), dietary habits (16 items), alcohol consumption and smoking (3 items), stress management (2 items), sleep and rest (3 items), and medication and health management (4 items). The answers ranged from 1 (rarely) to 4 (always). The possible score range was 36 to 144; higher scores reflected higher adoption of healthy lifestyle behaviors. The lifestyle evaluation tool used in this study had excellent internal consistency, reliability, and good validity [31]. In Kang’s study [31], the reliability of this tool was confirmed, with Cronbach’s α =0.92. In the present study, the lifestyle tool demonstrated good reliability (Cronbach’s α = 0.89).

Depression was measured using the Center for Epidemiologic Studies-Depression (CES-D), which was developed by the American Institute of Mental Health for community epidemiological investigations and was translated into Korean by Cho and Kim [32]. The CES-D was a 20-item screening tool designed to assess the frequency of perceived depressive symptoms over the past week. Items were rated on a 4-point Likert scale ranging from 0 (less than once a week) to 3 (more than 5 times a week). The possible score range was 0 to 60, and higher scores indicated more severe depression. In Cho and Kim’s study [32], the Korean version of the CES-D showed high internal reliability (Cronbach’s α = 0.93) and concurrent validity. The CES-D also showed good reliability in the present study (Cronbach’s α = 0.88)

Social support was measured using the Multidimensional Scale of Perceived Social Support (MSPSS), which was developed by Zimet, Dahlem, Zimet, Farley [33], and was translated into Korean by Shin and Lee [34]. The MSPSS was a tool for evaluating perceptions of the appropriateness of social support of family, friends, and other meaningful people. It is measured using a total of 12 questions, with 4 questions for each category of people who provided social support. The possible score range was 12 to 60; higher scores reflect higher perceived social support. When the MSPSS was developed, it showed good reliability (Cronbach’s α = 0.88) and construct validity. The MSPSS also showed excellent reliability in the present study (Cronbach’s α = 0.92).

### 2.3. Procedures

The data were collected from selected voluntary participants at each facility from 12 September 2019, through 15 November 2019. For each person who expressed interest in participating voluntarily in the study, the researchers or the designated staff at each facility distributed and explained the research protocol and obtained informed consent. The protocol stated that the collected data would not be used for purposes other than those set out in the form, that patients could withdraw from the study at any time, and that patient confidentiality would be ensured.

After learning about the purpose of the research, the participants were asked to complete 2 forms upon signing the consent form. Form 1 was designed to obtain information on general characteristics, medications taken by participants, and metabolic syndrome test results. The participants were asked to refer to their medication prescriptions and the results of metabolic syndrome tests conducted at the community health center or hospital. For those who indicated that it would be difficult for them to complete the form on their own, assistance was provided by researchers or the designated staff at each facility. Form 2 assessed lifestyle habits, depression, and social support, and participants were asked to fill out the form on their own. Each completed form was sealed in an envelope that was provided and collected, and a small cash reward was offered as compensation for their participation.

### 2.4. Statistical Analysis

Data analysis was performed using SPSS version 24.0 (IBM Corp, Armonk, NY, USA). The general characteristics of the participants, disease-related characteristics, lifestyle habits, psychosocial characteristics, and the prevalence of metabolic syndrome and each metabolic disorder were analyzed using descriptive statistics such as mean, standard deviation, number, and percentage. To assess the general characteristics, disease-related characteristics, lifestyle habits, and psychosocial characteristics according to the presence or absence of metabolic syndrome, the independent t-test, chi-square test, and Fisher’s exact test were used. Furthermore, to identify the risk factors of metabolic syndrome, multivariate logistic regression analysis was conducted using the variables found to show significant differences according to the presence of metabolic syndrome (employment status, body mass index, physical activity, and weight control, dietary habits, stress management, sleep and rest, medication and health management, depression, and social support). To determine the goodness of fit of the logistic regression model, the Hosmer–Lemeshow test was used.

### 2.5. Ethical Considerations

Data collection for this study was approved by the Ethics Committee of Kyung Hee University (approval no. KHSIRB-19-175).

## 3. Results

### 3.1. Prevalence of Metabolic Syndrome and Its Components

The prevalence of metabolic syndrome was 42.0%. Abdominal obesity (61.0%) was the most common characteristic of metabolic syndrome, followed by high plasma glucose (46.0%), high triglyceride levels (45.0%), low HDL-C levels (30.0%), and high blood pressure (28.0%) (Table 1).

### 3.2. Comparison of the Characteristics of General and Clinical Characteristics with and without Metabolic Syndrome

The mean age of the participants was 46.35 ± 11.54 years, and there were 52 men (52.0%) and 48 women (48.0%). The most common level of education was high school (64 patients, 64.0%) and the majority of participants (68 patients, 68.0%) were unmarried. Sixty-five participants (65.0%) were unemployed, and 82 participants (82.0%) had a monthly income of less than $2000. The participants’ mean body mass index was 26.06 ± 4.54 kg/m^2^; According to the standards of the Korean Society for Obesity, a normal weight was defined as a body mass index less than 23.0 kg/m^2^, overweight as 23.0–24.9 kg/m^2^, obesity class I as 25.0–29.9 kg/m^2^, obesity class II as 30.0–34.9 kg/m^2^, and obesity class III as 35.0 kg/m^2^ or more, respectively. In this study, slightly over half (56 patients, 56.0%) had a body mass index ≥ 25 kg/m^2^ (obesity class I or higher). The average duration of illness was 18.48 ± 10.20 years; it was 10 years or over in the majority of participants (79 patients, 79.0%). Atypical antipsychotics were used in most participants (60 patients, 60.0%), and antipsychotics were combined with antidepressants in 19 participants (19.0%).

Employment status (χ^2^ = 5.86, *p* = 0.015) and body mass index (χ^2^ = 13.40, *p* = 0.001) were general characteristics that showed statistically significant differences according to the presence or absence of metabolic syndrome. Clinical characteristics showed no statistically significant differences according to whether metabolic syndrome was present (Table 2).

### 3.3. Comparison of the Characteristics of Lifestyle and Psychosocial Factors with and without Metabolic Syndrome

Of the lifestyle factors, statistically significant differences according to the presence of metabolic syndrome were found for physical activity and weight control (t = 6.60, *p* < 0.001), dietary habits (t = 7.87, *p* < 0.001), stress management (t = 3.98, *p* < 0.001), sleep and rest (t = 3.64, *p* < 0.001), and medication and health management (t = 5.36, *p* < 0.001). Of the psychosocial characteristics, both depression (t = −5.18, *p* < 0.001) and social support (t = 3.36, *p* = 0.001) showed statistically significant differences according to the presence or absence of metabolic syndrome (Table 3).

### 3.4. Factors Influencing Metabolic Syndrome

To determine the risk factors for developing metabolic syndrome, multivariate logistic regression analysis was conducted with the following variables that showed significant differences depending on the presence or absence of metabolic syndrome: Employment status, body mass index, physical activity, and weight control, dietary habits, sleep and rest, stress management, medication and health management, depression, and social support. The risk of developing metabolic syndrome was affected by body mass index (*p* = 0.004, OR = 1.60), physical activity and weight control (*p* = 0.018, OR = 0.71), dietary habits (*p* = 0.011, OR = 0.72), medication and health management (*p* = 0.012, OR = 0.52, and depression (*p* = 0.008, OR = 1.22) (Table 4).

## 4. Discussion

The prevalence of metabolic syndrome in the study participants was 42.0%, which is similar to the rate of 46.5% found in people with schizophrenia in a previous study [3]. In a study that reviewed the prevalence of metabolic syndrome in the Asia-Pacific region, one-fifth of the general population was found to have metabolic syndrome [2]. In another study, the prevalence rate of metabolic syndrome in Korea was 20.3% between 2013 and 2015 [35]. Compared to the results of those two studies, the prevalence of metabolic syndrome among these community-dwelling schizophrenia patients was twice as high as that of the general population. Furthermore, the prevalence observed in this study was higher than has been observed in Japan (34.2%) [36], Taiwan (37.8%) [37], and Iran (23.9%) [38], which confirms the need for proactive management of metabolic syndrome in people with schizophrenia in Korea. Therefore, mental health care nurses who manage the health of people with schizophrenia living in the community need to recognize the high prevalence of metabolic syndrome among their patients and implement regular education and management.

In this study, logistic regression analysis was performed to determine risk factors for metabolic syndrome, using the independent variables that showed significant differences according to the presence of metabolic syndrome (employment status and body mass index, physical activity and weight control, dietary habits, stress management, medication and physical examination, depression, and social support). As a result, high body mass index and depression were confirmed to be associated with a higher risk of metabolic syndrome, whereas proper lifestyle habits related to physical activity and weight control, dietary habits, and medication and health management reduced the risk.

In this study, class I obesity or higher, defined as a body mass index of 25 kg/m^2^ or higher, was remarkably common (76.2%) among the participants with metabolic syndrome, which supports findings from previous studies suggesting that a high body mass index increases the risk of metabolic syndrome [3,37]. Body mass index is a key indicator of metabolic syndrome, and it is easily measurable and readily quantifiable [39]. A high body mass index indicates that an individual is obese, and obesity is not only associated with an increased risk of metabolic syndrome but is also a major risk factor of chronic diseases comorbid with high blood pressure, cerebrovascular disease, and diabetes [7,40]. In particular, compared to the general population, people with schizophrenia tend to have a higher body mass index and a three-fold higher risk of obesity [41]. Moreover, in order to manage the risk of metabolic disorder, patients taking antipsychotic agents are recommended to check their body mass index upon starting medication, and then to check their body mass index every month until 12 weeks have passed and every 3 months thereafter [42]. Thus, it is incumbent for mental health care nurses to routinely monitor the body mass index of people with schizophrenia in the community to manage or prevent metabolic syndrome.

In this study, depression was identified as a risk factor for metabolic syndrome in people with schizophrenia. In other words, the more depressed people with schizophrenia was, the more likely he or she was to develop metabolic syndrome. This finding supports the previous observation that depression was related to the development of metabolic syndrome in people with schizophrenia [43]. In general, depression stimulates the hypothalamic–pituitary–adrenal axis to increase the secretion of cortisol, which induces weight gain and dyslipidemia through the accumulation of visceral fat and inhibition of lipid mobilization [13]. Moreover, depressed mood causes reduced physical activity, increased food intake, drinking, and insufficient sleep, thereby increasing the risks of obesity and metabolic disorder [14,15]. The prevalence of comorbid depression is high (54.5% to 61.0%) in people with schizophrenia [16,17]. Therefore, a proactive approach is advised in managing metabolic syndrome in schizophrenia patients in the community; it is necessary to identify whether they have depressive symptoms, how severe the symptoms are, and whether they are taking antipsychotic agents, and on that basis, to provide aggressive interventions for depression [43].

In this study, among the lifestyle domains, proper physical activity and weight control, dietary habits, and medication and health management were found to reduce the incidence of metabolic syndrome. This is congruent with a previous report suggesting that poor dietary choices and lack of physical activity were associated with metabolic syndrome [44]. Dietary interventions designed to increase insulin sensitivity through the consumption of complex carbohydrates and unsaturated fatty acids were reported to reduce abdominal obesity through mechanisms such as reduction of adipose tissue mass and absorption of fat in the body [45]. In addition, according to a previous study that confirmed the relationship between dietary intervention and body mass index in patients with metabolic syndrome, healthy dietary habits such as a Mediterranean diet reduced body mass index and had an effect on the risk factors of metabolic syndrome, such as blood pressure, triglycerides, and high-density lipoprotein cholesterol [46]. Considering the finding in this study that a higher body mass index was associated with a higher risk of metabolic syndrome, good dietary habits themselves are expected to reduce the risk of metabolic syndrome directly, as well as indirectly, through the maintenance of a moderate body mass index. Besides, regular exercise both causes the reduction of visceral fat by promoting lipolysis through increased sympathetic nervous system activation and reduces the risk of metabolic syndrome by improving the blood lipid profile, blood pressure, and insulin resistance through promoting muscle mass accumulation and increasing fat oxidation [47]. Dietary and exercise interventions offered to people with schizophrenia were also reported to be effective in reducing weight, body mass index, and abdominal circumference and preventing metabolic syndrome and complications [48]. In a Spanish study involving patients with severe mental disorders in the community, 20 minutes of lifestyle education and 60 min of physical activity led to improvements in physical activity levels and health conditions in the experimental group [49]. In light of these findings, mental health care nurses should monitor the dietary habits and physical activity of their patients and develop and implement dietary and exercise programs that accommodate the characteristics of people with schizophrenia.

This study showed that regular medication use and health examinations were associated with a reduced risk of metabolic syndrome in people with schizophrenia. A previous study reported that awareness of a disease through health examinations motivated people to make lifestyle changes [50]. Moreover, the Information-Motivation-Behavior skill model, which was developed to assist in the self-management of patients with chronic diseases, suggests that sufficient information motivates patients and encourages them to make and maintain behavioral changes, which are expected to lead to health improvement [51]. Based on these studies, regular medication and health examination services for community-dwelling people with schizophrenia help them to obtain information about their own health status and adopt healthy lifestyle behaviors, ultimately reducing the prevalence of metabolic syndrome. However, in a recent study, 65.1% of community-dwelling people with schizophrenia had not received a fasting blood test in the most recent 2 years [25]. This low test rate seems to suggest that people with mental disorders may be less interested in physical health or lifestyle factors than the general population. However, recommendations from medical staff and guidance about testing have been reported to increase the screening rate of health examinations [25,52]. Therefore, in order to increase the frequency of health examinations in community-dwelling people with schizophrenia, mental health care nurses should recommend health examinations more actively and should systematically monitor changes in patients’ health status.

In this study, sex and age did not show statistically significant relationships with the presence or absence of metabolic syndrome. Previous studies involving the general population reported that the incidence of metabolic syndrome increased with age, and especially high incidence rates were found in men less than 50 years old and in women more than 50 years old [53]. However, in this study, the participants were people with schizophrenia aged between 18 and 65 at the time of inclusion in the study. Therefore, elderly individuals (over 65 years old), who would be necessary for a thorough comparison by age, were excluded. Instead, relatively young people (aged 40 or less) comprised 25% of all participants. This seems to explain why there was no significant difference in the prevalence of metabolic syndrome by age. Therefore, further research on the relationship between age and the risk of metabolic syndrome is needed, with appropriate participant selection considering age groups.

In this study, disease-related characteristics did not show statistically significant differences in the presence or absence of metabolic syndrome. These results differ from previous studies that reported that schizophrenia patients with metabolic syndrome had a longer prevalence than schizophrenia patients without metabolic syndrome and that olanzapine and clozapine were the major risk factors for developing metabolic syndrome [7,10,11]. This discrepancy may have occurred due to the fact that in this study, medications and dosage were only checked at the time of research, and it was difficult to verify the exact duration and dosage of medications that participants were taking at the community mental health care centers where data collection was conducted. Some participants had changed their medications depending on their symptoms in the most recent 6 months, and others had discontinued medications due to the incidence of chronic diseases such as diabetes and hypertension and changed to different antipsychotics. Therefore, in future research, it will be necessary to investigate the relationship between antipsychotics and metabolic syndrome in greater detail based on a careful review of data on types of antipsychotics, duration of taking the medications, and dosage.

This study has several limitations. First, the study included only people with schizophrenia who resided in Seoul and were registered with certain mental health welfare centers or mental rehabilitation facilities; thus, caution should be exercised in generalizing the results. Second, although the age range of the included patients (18–65 years) was wide enough to include a variety of age groups, due to the small number of subjects, it was difficult to compare differences in metabolic syndrome by age. Therefore, in future studies, it will be necessary to apply narrower inclusion criteria, focusing on those 40 years or over, as older individuals more frequently develop metabolic syndrome. Third, although we examined the duration of schizophrenia and the type of antipsychotic agents, other disease-related characteristics that may affect the occurrence of metabolic syndrome were not sufficiently investigated. Thus, we suggest that subsequent studies address the dosage and duration of antipsychotic treatment and the severity of negative symptoms, as well as the duration of illness and type of antipsychotics.

## 5. Conclusions

In this study, the prevalence of metabolic syndrome among people with schizophrenia living in the community was 42.0%, twice as high as that of the general population. High body mass index and depression were associated with an increased risk of metabolic syndrome, whereas proper lifestyle habits related to physical activity and weight control, diet, and medication and health management were associated with a lower risk of metabolic syndrome. Therefore, mental health care nurses need to recognize the high prevalence of metabolic syndrome in people with schizophrenia in the community and provide differentiated, customized lifestyle improvement programs based on the body mass index and depression status of each patient. Furthermore, comprehensive lifestyle improvement programs and health examination services that patients can easily adhere to should be developed.

## Figures and Tables

**Table 1:** Prevalence of metabolic syndrome and its components (*N* = 100).

Variables	Present	Absent	M(SD)
	*N* (%)	*N* (%)	
Metabolic syndrome	42(42.0)	58(58.0)	
Abdominal obesity(cm)	61(61.0)	39(39.0)	89.92(10.12)
High triglycerides(mg/dL)	45(45.0)	55(55.0)	152.26(77.76)
Low HDL(mg/dL)	30(30.0)	70(70.0)	49.48(9.85)
High plasma glucose(mg/dL)	46(46.0)	54(54.0)	103.41(19.44)
High blood pressure(mmHg)	28(28.0)	72(72.0)	118.87(14.45)

M = mean; SD = standard deviation; HDL = high density lipoprotein cholesterol.

**Table 2:** Comparison of the characteristics of study subjects with and without metabolic syndrome (*N* = 100).

Characteristics	Categories	*N* (%)	Non-MS (*N* = 58)	MS (*N* = 42)	χ^2^/t	*p*
			*N* (%)/M (SD)	*N* (%)/M (SD)		
**General**						
Gender	Man	52(52.0)	35(60.3)	17(40.5)	3.85	0.050
	Women	48(48.0)	23(39.7)	25(59.5)		
Age (years) Mean (SD) = 46.3 (11.54)	<40	25(25.0)	17(29.3)	8(19.0)	3.51	0.319
	40–49	32(32.0)	20(34.5)	12(28.6)		
	50–59	28(28.0)	15(25.9)	13(31.0)		
	≥60	15(15.0)	6(10.3)	9(21.4)		
Education level	≤Middle school	21(21.0)	10(17.2)	11(26.2)	1.60	0.450
	High school	64(64.0)	40(69.0)	24(57.1)		
	≥College	15(15.0)	8(13.8)	7(16.7)		
Marital status ^†^	Single	68(68.0)	43(74.1)	25(59.5)	2.50	0.275
	Married	8(8.0)	4(6.9)	4(9.5)		
	Divorced and others	24(24.0)	11(19.0)	13(31.0)		
Employed	Yes	35(35.0)	26(44.8)	9(21.4)	5.86	0.015
	No	65(65.0)	32(55.2)	33(78.6)		
Monthly income (US dollar)	<$2000	82(82.0)	45(77.6)	37(88.1)	1.82	0.177
	≥$2000	18(18.0)	13(22.4)	5(11.9)		
Body mass index(kg/m^2^) Mean (SD) = 26.0 (4.54)	<23	30(30.0)	25(43.1)	5(11.9)	13.40	0.001
	23–24.9	14(14.0)	9(15.5)	5(11.9)		
	≥25	56(56.0)	24(41.4)	32(76.2)		
**Clinical**						
Duration of mental illness (years) ^†^ Mean (SD) = 18.5 (10.20)	<5	7(7.0)	5(8.6)	2(4.8)	0.88	0.658
	5–10	14(14.0)	7(12.1)	7(16.7)		
	>10	79(79.0)	46(79.3)	33(78.5)		
Antipsychotics ^†^	None	12(12.0)	7(12.1)	5(11.9)	0.98	0.824
	Typical	9(9.0)	4(6.9)	5(11.9)		
	Atypical	60(60.0)	35(60.3)	25(59.5)		
	Combination	19(19.0)	12(20.7)	7(16.7)		
Antipsychotics with antidepressant	Yes	19(19.0)	12(20.7)	7(16.7)	0.26	0.613
	No	81(81.0)	49(79.3)	35(83.3)		

^†^ Fisher’s exact test; M = mean; SD = standard deviation; MS = Metabolic syndrome.

**Table 3:** Comparison of the characteristics of lifestyle and psychosocial factors with and without metabolic syndrome (*N* = 100).

Characteristics	Non-MS (*N* = 58)	MS (*N* = 42)	t	*p*
	M (SD)	M (SD)		
**Lifestyle**				
Physical activity and weight control	21.07(4.57)	14.76(4.91)	6.60	<0.001
Dietary habits	46.05(5.50)	35.36(7.48)	7.87	<0.001
Alcohol consumption and smoking	9.53(2.35)	8.69(3.16)	1.46	0.148
Stress management	9.02(2.27)	7.36(1.72)	3.98	<0.001
Sleep and rest	6.81(1.10)	5.86(1.52)	3.64	<0.001
Medication and health management	11.53(2.43)	8.81(2.62)	5.36	<0.001
**Psychosocial**				
Depression	13.22(8.21)	24.00(11.53)	−5.18	<0.001
Social Support	39.83(10.72)	32.17(11.94)	3.36	0.001

SD = Standard deviation; MS = Metabolic syndrome.

**Table 4:** Factors influencing metabolic syndrome (*N* = 100).

Characteristics	B	SE	OR	95%CI	*p*
**General**					
Employment status *	−0.07	1.05	1.07	0.14–8.37	0.949
Body mass index	0.47	0.16	1.60	1.16–2.18	0.004
**Lifestyle**					
Physical activity and weight control	−0.34	0.14	0.71	0.54–0.94	0.018
Dietary habits	−0.33	0.13	0.72	0.54–0.93	0.011
Sleep and rest	−0.42	0.55	0.66	0.23–1.94	0.449
Stress management	−0.38	0.29	0.68	0.39–1.20	0.186
Medication and health management	−0.66	0.26	0.52	0.31–0.86	0.012
**Psychosocial**					
Depression	−0.20	0.08	1.22	1.06–1.42	0.008
Social support	−0.06	0.05	0.95	0.85–1.05	0.279

B = Regression coefficient; SE = Standard error; OR = Odds ratio; CI = Confidence interval; * Dummy variable: Employment (yes = 1, no = 2).
